# Pulmonary Toxicity of Boron Nitride Nanomaterials Is Aspect Ratio Dependent

**DOI:** 10.1021/acsnano.3c06599

**Published:** 2023-12-05

**Authors:** Luis Augusto Visani de Luna, Thomas Loret, Yilin He, Morgan Legnani, Hazel Lin, Anne Marie Galibert, Alexander Fordham, Sonja Holme, Antonio Esau Del Rio Castillo, Francesco Bonaccorso, Alberto Bianco, Emmanuel Flahaut, Kostas Kostarelos, Cyrill Bussy

**Affiliations:** † Nanomedicine Lab, Faculty of Biology, Medicine and Health, The University of Manchester, Manchester Academic Health Science Centre, Manchester M13 9PT, U.K.; ‡ National Graphene Institute, The University of Manchester, Manchester, M13 9PL, U.K.; § Lydia Becker Institute of Immunology and Inflammation, Faculty of Biology, Medicine and Health, The University of Manchester, Manchester Academic Health Science Centre, Manchester M13 9PT, U.K.; ∥ CNRS, Immunology, Immunopathology and Therapeutic Chemistry, UPR 3572, University of Strasbourg, ISIS, 67000 Strasbourg, France; ⊥ CIRIMAT, Université Toulouse 3 Paul Sabatier, Toulouse INP, CNRS, Université de Toulouse, 118 Route de Narbonne, 31062 Toulouse cedex 9, France; # BeDimensional S.p.A., Lungo Torrente Secca 30r, 16163 Genoa, Italy; ∇ Istituto Italiano di Tecnologia, Graphene Laboratories, Via Morego 30, 16163 Genoa, Italy; 8 Catalan Institute of Nanoscience and Nanotechnology (ICN2), CSIC and BIST,, Campus UAB, Bellaterra, 08193 Barcelona, Spain

**Keywords:** 2D materials, nanotubes, lungs, clearance, inflammation, fibrosis, genotoxicity

## Abstract

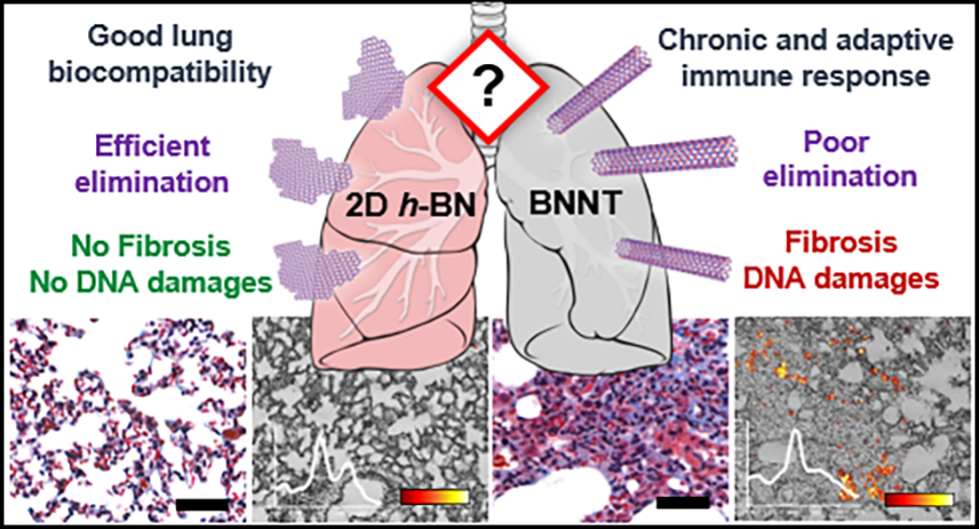

Boron nitride (BN) nanomaterials have drawn a lot of interest in the material science community. However, extensive research is still needed to thoroughly analyze their safety profiles. Herein, we investigated the pulmonary impact and clearance of two-dimensional hexagonal boron nitride (*h*-BN) nanosheets and boron nitride nanotubes (BNNTs) in mice. Animals were exposed by single oropharyngeal aspiration to *h*-BN or BNNTs. On days 1, 7, and 28, bronchoalveolar lavage (BAL) fluids and lungs were collected. On one hand, adverse effects on lungs were evaluated using various approaches (*e.g*., immune response, histopathology, tissue remodeling, and genotoxicity). On the other hand, material deposition and clearance from the lungs were assessed. Two-dimensional *h*-BN did not cause any significant immune response or lung damage, although the presence of materials was confirmed by Raman spectroscopy. In addition, the low aspect ratio *h*-BN nanosheets were internalized rapidly by phagocytic cells present in alveoli, resulting in efficient clearance from the lungs. In contrast, high aspect ratio BNNTs caused a strong and long-lasting inflammatory response, characterized by sustained inflammation up to 28 days after exposure and the activation of both innate and adaptive immunity. Moreover, the presence of granulomatous structures and an indication of ongoing fibrosis as well as DNA damage in the lung parenchyma were evidenced with these materials. Concurrently, BNNTs were identified in lung sections for up to 28 days, suggesting long-term biopersistence, as previously demonstrated for other high aspect ratio nanomaterials with poor lung clearance such as multiwalled carbon nanotubes (MWCNTs). Overall, we reveal the safer toxicological profile of BN-based two-dimensional nanosheets in comparison to their nanotube counterparts. We also report strong similarities between BNNTs and MWCNTs in lung response, emphasizing their high aspect ratio as a major driver of their toxicity.

## Introduction

Two-dimensional (2D) hexagonal boron nitride (*h*-BN) nanosheets and one-dimensional (1D) boron nitride nanotubes (BNNTs) are often compared to carbon nanomaterials. On the one hand, *h*-BN nanosheets resemble graphene due to their similar molecular organization in a honeycomb lattice, although with boron and nitrogen atoms replacing carbon in the aromatic condensed structure.^1^ On the other hand, BNNTs present structural similarities to multiwalled carbon nanotubes (MWCNTs). Just like carbon nanomaterials, *h*-BN nanosheets and BNNTs have distinct properties, including mechanical resistance, thermal stability, electrical resistance, and a high surface adsorption capability that could be useful in a wide range of applications from aerospace to nanomedicine.^2−9^

Although they possess exceptional properties, there are still concerns about their safety profiles, particularly when it comes to unintentional inhalation during production, processing, and inappropriate powder handling. Previous studies have already highlighted the potential toxicity of BNNTs.^10,11^ For instance, Kodali et al. have shown that BNNTs and MWCNTs caused similar acute responses *in vivo* and *in vitro*.^10^ In a follow-up study, Xin et al. reported lung inflammation up to one month after exposure to BNNTs of 50 wt % purity (the remaining 50 wt % was other forms of boron nitride structures).^11^ In this report, a strong immune cell infiltration in the form of granulocytes and lymphocytes was measured.^11^ Such effects may be attributed to the poor clearance of the materials because 50% of the BNNTs were still present in the lungs after 2 months. However, the authors did not report any tissue remodeling or fibrosis. The absence of fibrosis is in contrast to what was reported for MWCNTs, which are known to cause chronic inflammation and permanent tissue damage, including fibrosis, DNA damage, and cancer, when they are long and rigid.^12−15^ However, this lack of long-term adverse outcomes may be attributed to the purity of the materials because only 50 wt % of materials administrated were made of nanotubes; hence half of the dose was not made of nanotubes. Overall, it remains unknown if BNNTs with a greater amount of nanotubes per mass could cause permanent lung damage at doses similar to those used in previous *in vivo* works^10,11^ or at a lower doses representative of occupational exposure.^16^

The safety profile of *h*-BN nanosheets remains also largely unknown due to the lack of *in vivo* studies. Recently, Lucherelli et al., investigated two types of *h*-BN nanosheets, one with a cornered/sharp structure and one with a round form, the latter similar to *h*-BN used in the present study.^17^ The cornered *h*-BN nanosheets have shown dose-dependent cytotoxicity to lung epithelial H460 cells, while the round *h*-BN did not provoke any significant cell death.^17^ The combination of molecular dynamics simulations and *in vitro* testing demonstrated that the sharp *h*-BN edges were able to pierce the lipid membrane, leading to the formation of water channels, provoking membrane permeabilization of lysosomes, the release of cathepsin B, and generation of radical oxygen species, all of which was leading to cell apoptosis.^17^ Therefore, this study highlighted that the shape of 2D materials is an important physicochemical parameter influencing the biological interaction with cells.^17^ In another *in vitro* study using the macrophage-derived THP-1 human cell line, Kodali et al., have shown that both BNNTs and *h*-BN induced biological responses 24 h after exposure.^18^ They found that *h*-BN and BNNTs caused significant increases in the secretion of several pro-inflammatory cytokines, including IL-1β and GM-CSF with some of them secreted in response to BNNTs, but not to *h*-BN.^18^ They reported a significant activation of the NLRP3 inflammasome only after exposure to BNNTs and established the lowest observed adverse effects level (LOAEL) for cytokine secretion at 25 μg/mL for purified BNNTs and at 100 μg/mL for *h*-BN.^18^ They also found less cytotoxicity for *h*-BN and impure BNNTs (low amount of nanotubes per mass) than for pure BNNTs (high amount of nanotubes per mass).^18^

Observing less toxicity for low aspect ratio *h*-BN nanosheets in comparison to high aspect ratio BNNTs agrees with the fiber pathogenicity paradigm and definition of high aspect ratio nanomaterials. It is well accepted that high aspect ratio materials, with a length above 5 μm and a ratio “length-diameter/thickness” greater than 3, could cause chronic lung toxicity and permanent damage after lung administration if they reached the alveolar region and induced frustrated phagocytosis in alveolar macrophages.^19−22^ This paradigm has been established and confirmed through an extensive number of studies, notably on carbon nanotubes and asbestos.^19,23^ In recent works, some of us tested whether graphene-based materials (GBMs) with large lateral dimensions (>5 μm) and high aspect ratio could cause similar pulmonary adverse effects as long carbon nanotubes.^24−27^ GBMs were found to be relatively safe in comparison to MWCNTs. Although large graphene oxide (GO) sheets (>5 μm) caused biological effects typically reported for high aspect ratio materials, including frustrated phagocytosis and the entrapment of materials in granulomatous-like clusters, there was no permanent damage, unlike what was observed for long MWCNTs.^26^ We concluded that the absence of adaptive immune activation was a key feature that prevents progression toward pathological conditions in the case of the large GO sheets, which like MWCNTs were considered as high aspect ratio materials despite their 2D nature.^26^

By extension, we hypothesized that the high aspect ratio nanomaterials and fiber pathogenicity paradigms would both apply to BNNTs but not to *h*-BN nanosheets, which here qualify as low aspect ratio materials due to their small lateral dimensions. To test this hypothesis, we exposed mice by single oropharyngeal aspiration to *h*-BN nanosheets and BNNTs and tested both the fate and the impact of these materials in the lungs.

## Results and Discussion

### Characterization and Clearance from the Lungs of *h*-BN and BNNTs

#### Synthesis and Characterization of the Boron Nitride Materials

**Figure 1:**
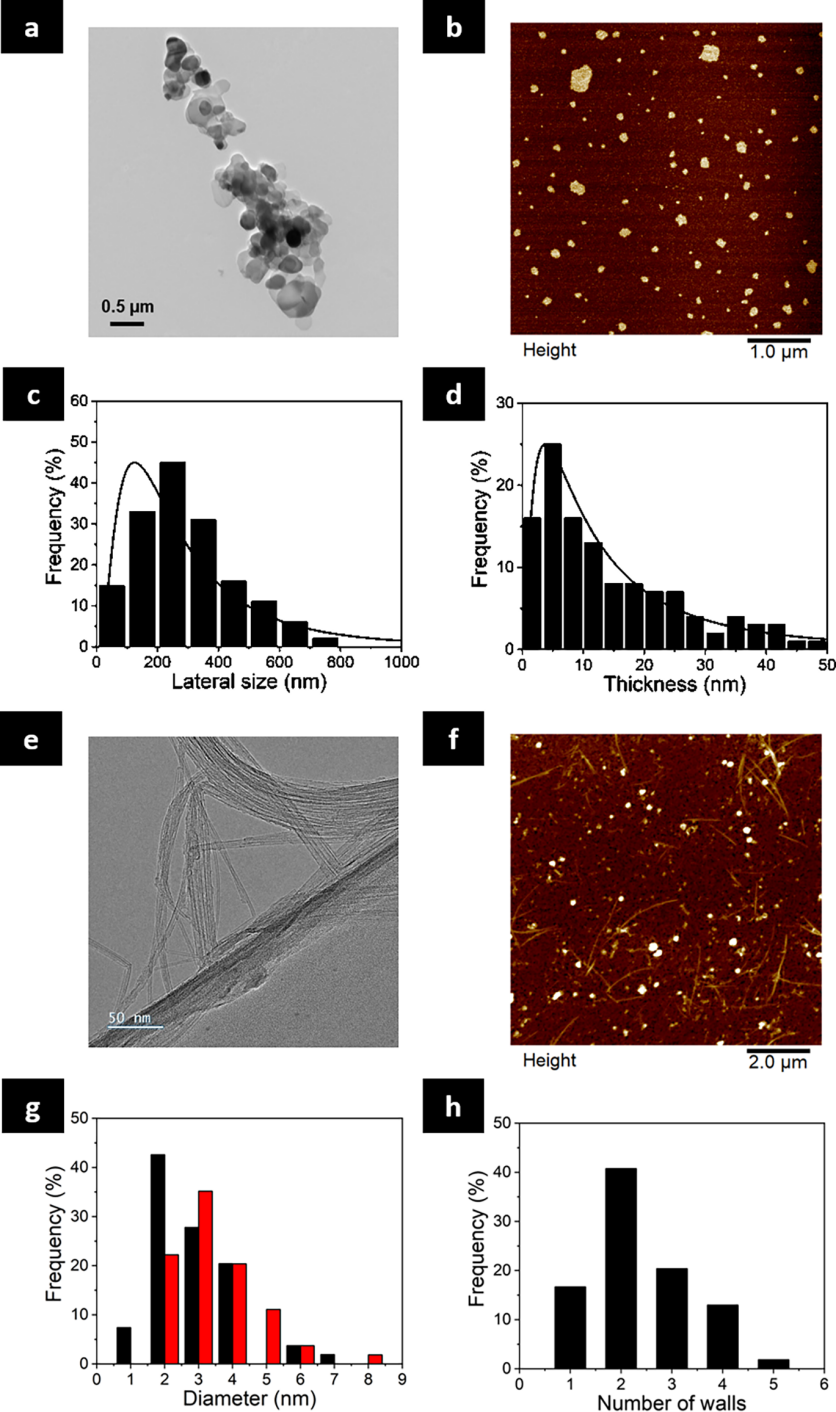
Structural characterization of *h*-BN and BNNTs. (a) TEM image of *h*-BN. (b) AFM image of *h*-BN. (c,d) Lateral size (LogNorm. SD 0.797, *N* = 159) and thickness distributions (LogNorm. SD 1.054, *N* = 129) of *h*-BN nanosheets. (e) TEM image of BNNTs. (f) AFM image of BNNTs. (g) Diameter distribution of walls for BNNTs (black and red bars correspond to internal and external diameters, respectively). (h) Number of walls for BNNTs.

Two-dimensional hexagonal boron nitride nanosheets were prepared through liquid phase exfoliation of bulk *h*-BN in water and sodium cholate, used as a surfactant.^28,29^ The morphological analysis was assessed by transmission electron microscopy (TEM) and atomic force microscopy (AFM) analyses. Figure 1A shows that the exfoliated *h*-BN nanosheets consisted of round-shaped flakes with a size distribution between 3.8 and 750 nm, 50% of the nanosheets in the range 100–400 nm, and a lateral size mode of 290 nm (Figure 1C). The AFM images of individual nanosheets (Figure 1B) give a hint about the flake thickness distribution, showing a mode of 3.7 nm (Figure 1D). Additionally, Raman spectroscopy confirms the crystalline nature of the *h*-BN, showing the characteristic *E*_2*g*_ phonon mode at ∼1370 cm^–1^, Supporting Information (SI), Figure S1A.^30^ The chemical composition was then analyzed by X-ray photoelectron spectroscopy (XPS) (SI, Figure S2A), in which B 1s and N 1s spectra of *h*-BN displayed the expected binding energy of the main peaks at 190.4 and 398.0 eV, respectively (SI, Figure S2B and S2C). The atomic percentage present in *h*-BN nanosheets was calculated from the XPS survey (SI, Table S1), showing the presence of boron, nitrogen, carbon, and oxygen (C and O were attributed to sodium cholate). Finally, the presence of sodium cholate was confirmed by thermogravimetric analysis (TGA), displaying a first weight loss of ∼8%, associated with the absorbed sodium cholate on the surface of nanosheets (SI, Figure S1C).

The BNNTs (refined puffballs #SP10RX; with less than 1% elemental boron according to supplier) were obtained from BNNT LLC (USA) and dispersed in ethanol using bath ultrasonication, followed by a solvent exchange to obtain a final homogeneous dispersion in water. The high-resolution TEM (HR-TEM) (Figure 1E) and AFM (Figure 1F) images of BNNTs evidenced bundles of tubular structures with a diameter distribution between 1 and 7 nm for the inner diameter and between 2 and 8 nm for the outer diameter (Figure 1G). These nanotubes had between 1 and 4 walls, with a predominance of double-walled BNNTs (Figure 1H). The length of the BNNTs was challenging to measure due to entanglement, but it was determined to be longer than 5 μm and up to a few tens of μm when gathered into bundles, in agreement with AFM images and supplier information. Moreover, Raman spectroscopy analysis revealed that BNNTs displayed the representative sharp peak located at ∼1370 cm^–1^, indicating the crystallinity of the materials (SI, Figure S1B).^30^ In XPS analysis, B1, N1, C1, and O 1s were identified (SI, Figure S2D and Table S1), with C and O attributed to the carbon tape used for the sample preparation. Similarly, the binding energies in the high-resolution B 1s and N 1s spectra of BNNTs were located at 190.6 and 398.4 eV, respectively (SI, Figure S2E and S2F). In addition, the atomic percentage present in BNNTs was calculated from the XPS survey (SI, Table S1). The TGA profile of BNNTs shows a weight loss of only 2%, accounting for the good thermal stability of BNNTs (SI, Figure S1D). In agreement with the data provided by the supplier, it was determined that 80–90% of the BNNT material was made of tubes, present as either individualized tubes or bundles.

#### Evaluation of h-BN and BNNT Elimination from Lung Tissues Using Raman Spectroscopy

**Figure 2:**
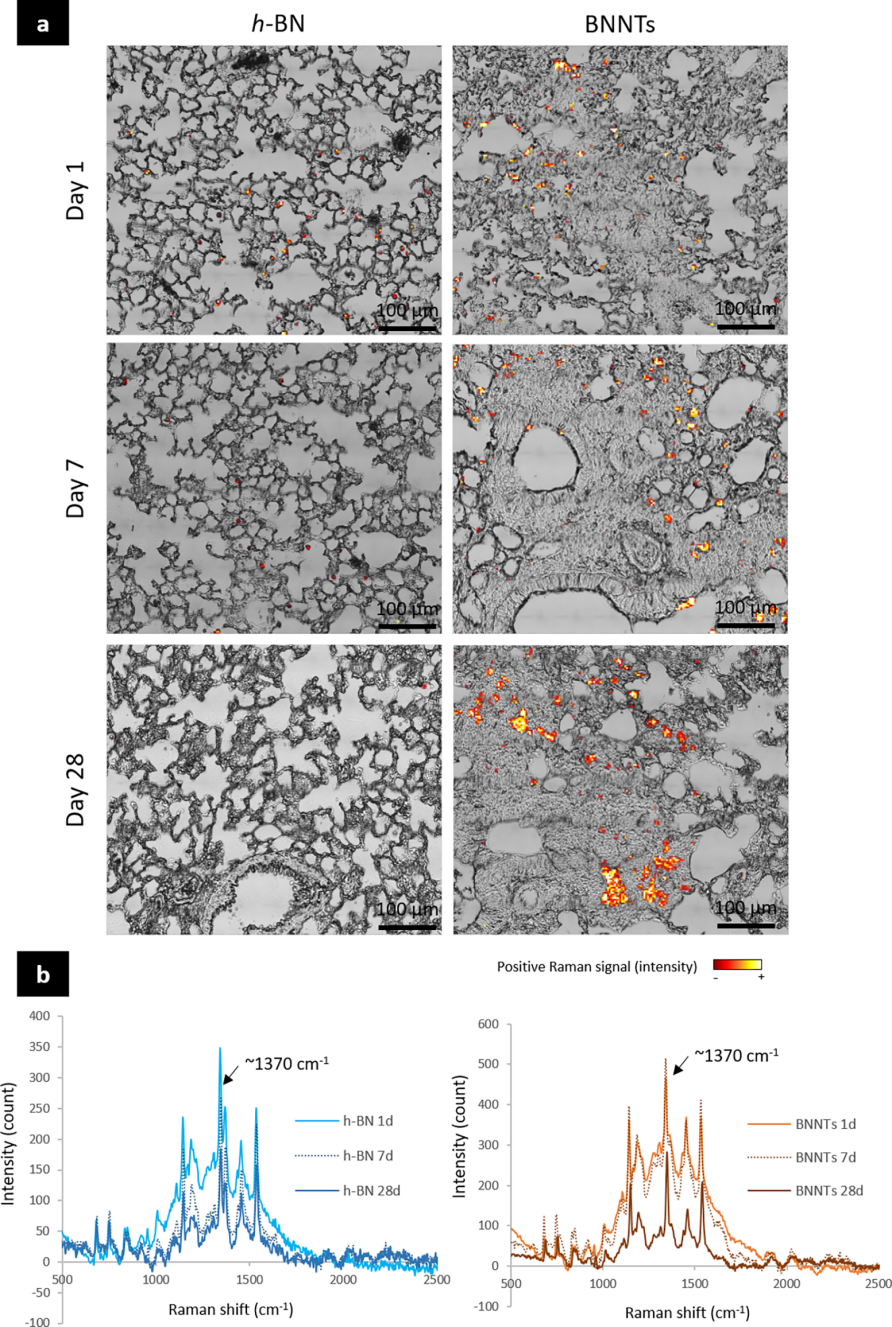
Evaluation of boron nitride distribution and clearance using scanning Raman spectroscopy. Mice were exposed by single oropharyngeal aspiration to 30 μg of *h*-BN or BNNTs. One, 7, and 28 days after the last exposure, left lungs were collected, inflated with formalin, embedded in paraffin, and then processed (*n* = 5). Lung sections (5 μm) were dewaxed and scanned using scanning Raman spectroscopy. *h*-BN and BNNTs were identified based on the Raman shift at 1370 cm^–1^. (a) Overlay of Raman intensities and bright-field images are presented. (b) Variation of boron (1370 cm^–1^) intensity over time.

On days 1, 7, and 28 after administration of the BN materials in mice by oropharyngeal aspiration (*i.e.*, 30 μg/animal), lung tissues were harvested, sectioned, and scanned by Raman spectroscopy to evaluate *h*-BN and BNNT presence in the lung tissue sections. Both *h*-BN and BNNTs were detected in the lungs (Figure 2). When overlaying the Raman signatures (boron nitride nanomaterials peak at ∼1370 cm^–1^) and the bright-field images (Figure 2), we found a clear interaction between *h*-BN nanosheets and macrophages from day 1, suggesting a rapid internalization of the materials. The Raman signature associated with *h*-BN was identified in these cells up to 7 days after exposure, while fewer materials (*i.e*., fewer Raman counts) were detected on day 28, suggesting an efficient elimination from the lungs over time, likely by macrophages. On the contrary, BNNTs were observed to be persisting in the lungs over time, as evidenced by a high number of positive Raman signals detected up to day 28. In the overlay pictures, the BNNTs appeared entrapped in denser tissue areas and tended to form material agglomerates that increased in size with time (*i.e.*, higher Raman counts), suggesting poor clearance from the lungs (Figure 2).

Additionally, changes in Raman signatures (variation in peaks’ shape) and intensities over time were considered as indications of biodegradation. In addition to the typical Raman shift at about 1370 cm^–1^, we found other unidentified peaks as well as an overall increased intensity from 1000 to 1800 cm^–1^, that could resemble the signature of amorphous boron nitride.^31^ This phenomenon may also be attributed to agglomeration after internalization in phagocytes. Nevertheless, we could still observe clear decreases in Raman signal intensities for *h*-BN nanosheets, from day 1 to day 7 and then from day 7 to 28, but only from day 7 to 28 for BNNTs. The decreases in intensity suggested that both BN materials could be degraded *in vivo*, as previously demonstrated *in vitro* using various oxidative conditions, including myeloperoxidase, which is found in the phagolysosome of macrophages.^32^ Moreover, the differences between *h*-BN and BNNTs (i.e., regular changes in Raman signature for *h*-BN at each tested time point; appearing only at later stage for BNNTs) highlighted that *h*-BN nanosheets might be degraded faster than BNNTs.

In summary, *h*-BN nanosheets had their highest accumulation at day 1 because they were eliminated regularly from the lungs over time (either *via* cell-mediated removal or via degradation), while BNNTs were persistently present at each of the time points tested with no apparent or limited evidence of elimination. The formation of BNNT agglomerates that increased in size with time, alongside the changes in tissue morphology with time as seen in the bright field picture (see further details in the Histological Changes section below), strongly suggested that these materials were inducing biological changes and tissue remodeling leading to the gathering of materials into material-cell structures that could be shielded from the rest of the lung parenchyma. Overall, these findings suggested that BNNTs would likely have stronger adverse effects than *h*-BN because poor clearance and slower material degradation from the lungs have often been associated with increased toxicity and tissue damage.^21,22,26,27^

### Boron Nitride Nanomaterials Induced Inflammation Is Aspect Ratio Dependent

#### Impact on BAL Cell Populations

**Figure 3:**
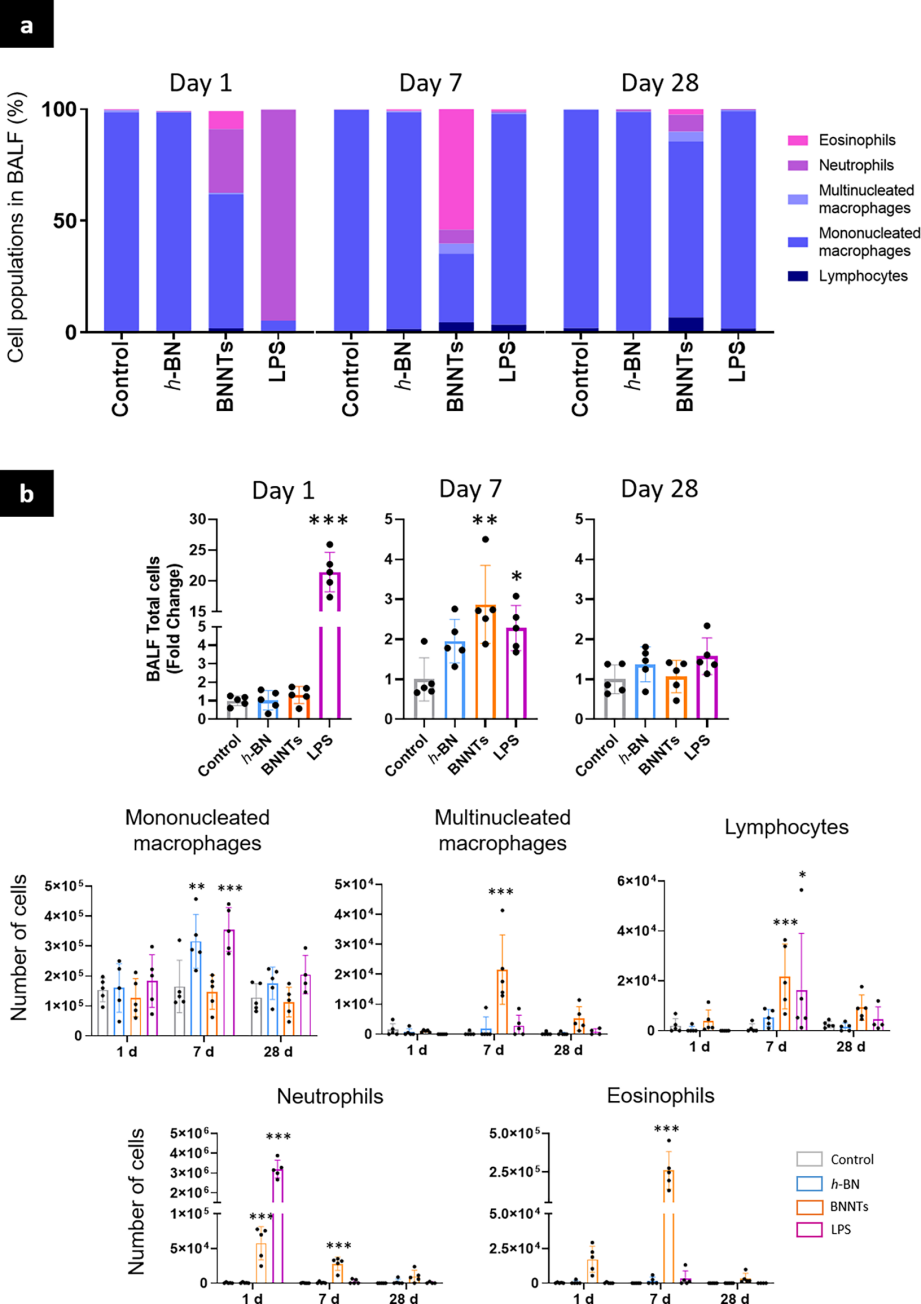
Influx of inflammatory cells in alveolar space. Mice were exposed by oropharyngeal aspiration to 30 μg of *h*-BN, BNNTs, or to control (vehicle and LPS). BALFs were collected on days 1, 7, and 28, cytospun on slides, and then differentially stained for cell phenotyping. (a) Cell population in BALFs. (b) Total number of immune cells. (c) Number of mononucleated macrophages, multinucleated macrophages, neutrophils, eosinophils, and lymphocytes in BALF. Two-way ANOVA followed by Tukey’s multiple comparisons test was used to evaluate statistical differences compared to the negative control (*p* < 0.05 (*), *p* < 0.01 (**), *p* < 0.001 (***)).

The immune cells present in BAL fluids (BALFs) after exposure to BN nanomaterials were then identified using differential staining (Figure 3a–c, and SI, Figures S3). Overall, low aspect ratio *h*-BN nanosheets did not significantly change the total number of BAL immune cells at any time point, while high aspect ratio BNNTs induced a significant increase in the total number of immune cells at day 7 (Figure 3b).

However, when going into individual cell types (Figure 3c), we noted a significant increase in the number of mononucleated macrophages 7 days after exposure to *h*-BN nanosheets. This can be attributed to the recruitment of circulating macrophages in order to support/complement the lung resident alveolar macrophages toward clearing *h*-BN nanosheets from the alveoli. At day 28, once the materials were removed from the alveoli, as suggested by the Raman spectroscopy imaging data (Figure 2), the levels of macrophages were similar to those found for the negative control group.

For BNNTs, there was a significant influx of neutrophils on day 1, and neutrophils were still present on day 7, although decreasing in number in comparison to day 1 (Figure 3c). By day 28, neutrophil levels were almost back to the negative control level. Eosinophil levels, while not significantly increased on day 1 in BNNT-exposed animals, displayed a statistically significant increase on day 7, but were similar to those of the negative control on day 28. Lymphocytes were also detected in BALF of BNNT-exposed animals, with a statistically significant increase in the number recorded on day 7, resolving to nonsignificant level on day 28, albeit still above negative control. This increase in lymphocytes of BNNT-exposed animals suggested a potential activation of adaptive immunity. Additionally, the significant increase in multinucleated macrophages on day 7 in BNNT-exposed animals (Figure 3c), suggested an ongoing frustrated phagocytosis, eventually leading to granulomatous structures (see histological changes section below), which has been documented previously for other high aspect ratio nanomaterials and linked to their poor clearance from the lungs.^26^ This increase in the number of multinucleated macrophages is typically attributed to the fusion of mononucleated macrophages that are struggling to eliminate the materials from the lungs. By fusing, mononucleated macrophages tend to gather the materials they were attempting to engulf, leading to an increase in material agglomerates over time, in line with what Raman imaging was suggesting (Figure 2). By day 28, the number of multinucleated macrophages in BALF albeit above the negative control was no longer significantly different (Figure 3c). However, this apparent resolution of inflammation over time after BNNT exposure (*i.e*., immune cells were no longer recruited to the BALF on day 28; Figure 3b,c) does not exclude that the recruited immune cells were still in the airways, but while not in the BALF, they were contributing to granulomas that tend to be strongly attached to the lung parenchyma *via* their action on extra cellular matrix remodeling. Such formation of multinucleated macrophages in BALF leading to granulomas in lung tissue sections after frustrated phagocytosis of materials has been reported before for other high aspect ratio nanomaterials and is typically due to the biopersistence and limited elimination of those materials from the lungs.^26^ The present biological results are therefore in agreement with the persistence and agglomeration of materials revealed by scanning Raman spectroscopy (Figure 2), which suggested limited elimination of the BNNTs from the lungs.

#### Expression of Inflammatory Markers in Lungs

**Figure 4:**
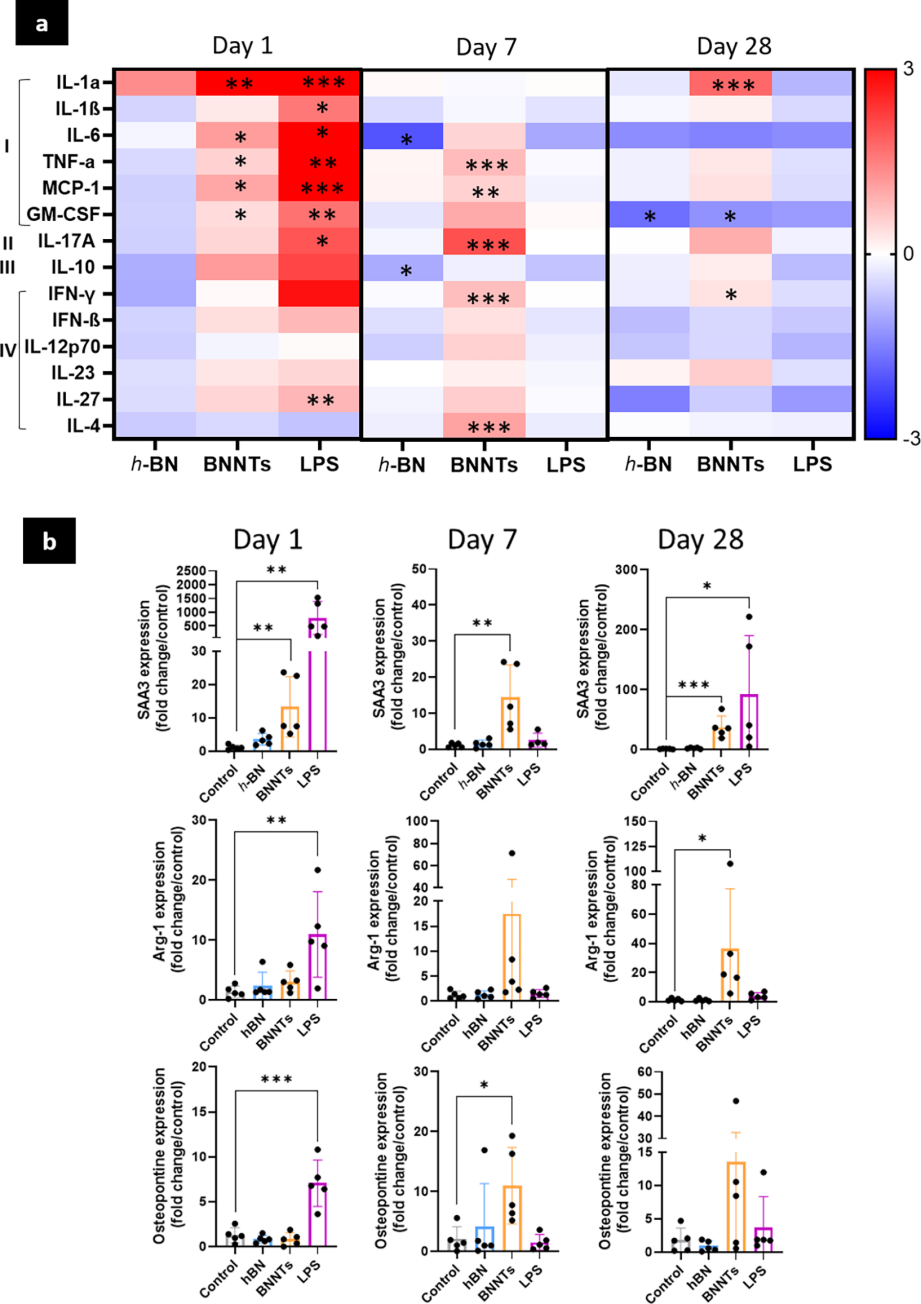
Inflammatory response in lungs. (a) Evaluation of inflammatory markers levels in lungs by multiplex ELISA (log2 fold change). (I) Acute inflammation; (II) late pro-inflammatory response; (III) anti-inflammatory response; (IV) adaptive immune activation. (b) Evaluation of SAA3, Arg-1, and osteopontin levels by RT-qPCR. One-way ANOVA followed by Dunnett posthoc test was used to evaluate any statistical difference between normalized cytokine concentrations (pg/mg of total protein measured with Pierce Assay) or fold change compared to the negative control (*n* = 5; (*) *p* < 0.05, (**) *p* < 0.01, (***) *p* < 0.001).

Exposure of mice to *h*-BN nanosheets by oropharyngeal aspiration did not induce any significant pulmonary inflammation, as shown in Figure 4. None of the protein markers measured by multiplex enzyme-linked immunosorbent assay (ELISA) were secreted in significant amounts or upregulated in comparison to the control (Figure 4a,b). This is not surprising because we did not find any influx of granulocytes in BALF after exposure to *h*-BN nanosheets. Moreover, the absence of significant upregulation of serum amyloid A3 (SAA3), which is a marker of acute response in the lungs,^26,33,34^ also supports the absence of a clear inflammatory response for the low aspect ratio *h*-BN (Figure 4b). Additional markers, including IL-6 and IL-10 on day 7 and GM-CSF on day 28, decreased in comparison to the control (Figure 4a). There was also no upregulation of Arg-1 and osteopontin (Figure 4b), which are typically expressed in the lungs in response to inflammation,^26^ supporting the absence of significant anti-inflammatory activation.

Overall, the results obtained for low aspect ratio *h*-BN were in clear contrast to those found for high aspect ratio BNNTs. The nanotubes caused a significant increase in several pro-inflammatory cytokines, including IL-1α on days 1 and 28, IL-6 on day 1, and TNF-α and MCP-1 on days 1 and 7 (Figure 4a). We also measured an increase in GM-CSF on days 1 and 7 (significant only on day 1). Additionally, significant increases in cytokines linked to an adaptive immune response were noted, including IFN-γ on days 7 and 28 and IL-4 on day 7, underlining the potential activation of both Th1 and Th2 responses, as the increase in lymphocyte number in BALF at these two time points could suggest (Figure 3). Increases in IL-27 and IL-17 on days 1 and 7 were also indicative of adaptive immunity activation (Figure 4a) because these two cytokines are involved in lymphocyte activation pathways. Moreover, the clear upregulation of the acute phase response protein SAA-3 at any time points tested after exposure to BNNTs further confirmed that these high aspect ratio materials induced both acute and chronic lung inflammation (Figure 4b). However, we did not measure any significant increase in the SAA blood content of the BNNT-exposed animals (SI, Figure S4), suggesting that the inflammation may remain localized to the lungs and was not systemic at the tested time points after a single exposure. Finally, we found a clear upregulation of both Arg-1 and osteopontin on days 7 and 28 in response to the ongoing inflammation (Figure 4b). Altogether, the evidence of chronic inflammation and activation of adaptive immunity for BNNTs suggests that long-term damage may arise in the lungs and other organs, as previously shown after exposure to other high aspect ratio materials, such as MWCNTs.^20,26,35,36^ The consistent activation of SAA3 after a single exposure, a biomarker often linked to the risk of cardiovascular diseases,^37,38^ further stresses the need to study the impact of repeated exposure to BNNTs beyond the lungs and in particular on the cardiovascular system.

### Nanotubes but Not 2D Nanosheets Caused Lung Tissue Damage

#### Histological Changes

**Figure 5:**
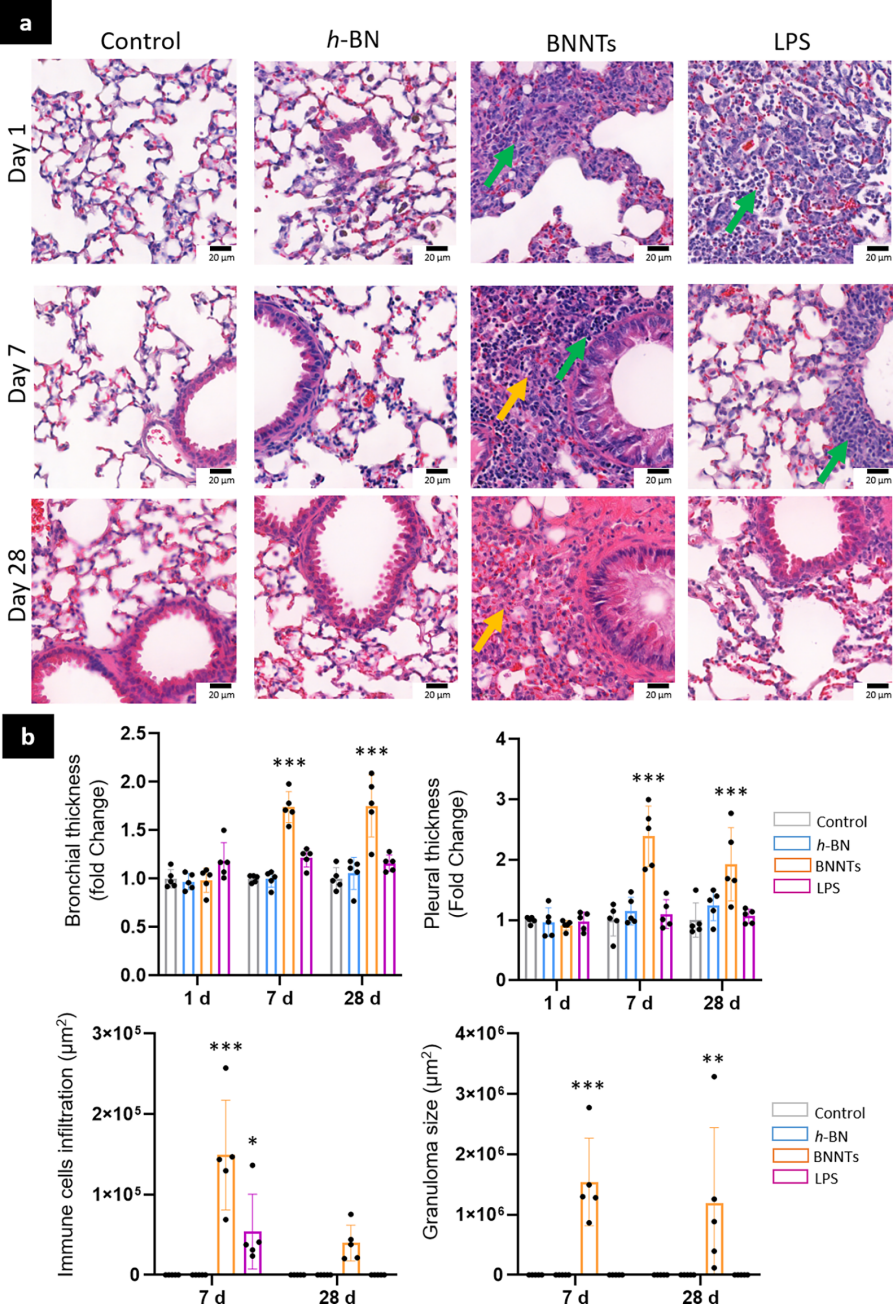
Histopathological changes in lungs. Mice were exposed to *h*-BN, BNNTs, or controls. (a) Lung sections were stained in hematoxylin and eosin for histopathological analysis. (b) Bronchial and pleural thicknesses were recorded. Immune infiltrates (green arrow), and granulomatous-like structures (yellow arrow) were identified and measured. Two-way ANOVA followed by Tukey’s multiple comparisons test was used to evaluate statistical differences between materials exposure and the negative control (*n* = 5; *p* < 0.05 (*), *p* < 0.01 (**), *p* < 0.001 (***)).

Tissue damage induced by *h*-BN and BNNT materials was evaluated on lung sections stained with hematoxylin and eosin. The bronchial thickness, pleural thickness, lymphocyte infiltration, and formation of granulomatous structures were recorded (Figure 5). As indicated by the inflammation results mentioned earlier (Figures 3 and 4), exposure to *h*-BN did not cause any significant influx of immune cells or noticeable tissue damage in the exposed animals (Figure 5a,b). However, exposure to high aspect ratio BNNTs caused a significant increase in both bronchial and pleural thicknesses on days 7 and 28. There was also significant immune cell infiltration, including lymphocytes, in the lungs of BNNT-treated mice (green arrow) as suggested by the BALF analysis. Finally, the presence of granuloma-like formations in the lung parenchyma (indicated by the yellow arrow) was deemed statistically significant on days 7 and 28. Although a decrease in immune cell infiltration and granuloma sizes was measured as time progressed, these granulomatous structures were still present on day 28, suggesting a potential chronic impact of BNNTs as well as persistent tissue damage.

#### Evaluation of Fibrosis and Potential DNA Damage in the Lung Parenchyma

**Figure 6:**
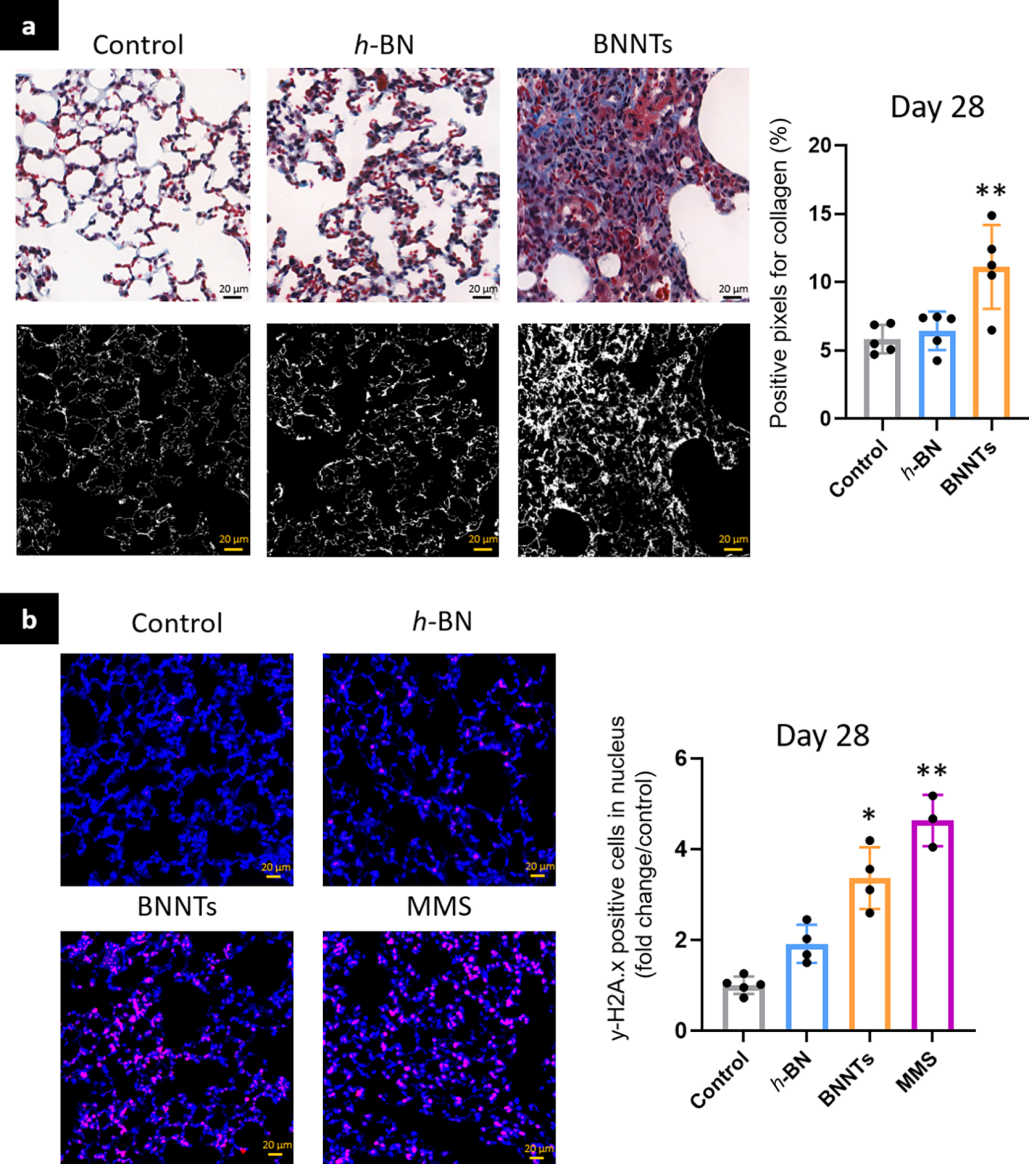
Fibrosis and DNA damage in the lungs. Mice were exposed to *h*-BN, BNNTs, or controls; lungs were collected after 28 days. (a) Lung sections were stained with Masson’s Trichrome, then collagen deposition was evaluated using MATLAB. (b) Lung sections from day 28 were immunostained for γ-H2AX to evaluate DNA double-strand breaks and potential long-term genotoxicity. One-way ANOVA followed by Dunett’s posthoc test was used to evaluate statistical differences between *h*-BN or BNNTs and the negative control (*n* = 5; *p* < 0.05 (*), *p* < 0.01 (**), *p* < 0.001 (***)).

Lung fibrosis is characterized by the permanent deposition of collagen in the parenchyma and interstitial lung. Lung sections of mice exposed to *h*-BN and BNNTs were therefore stained using Masson’s Trichrome to evaluate collagen deposition in the lung parenchyma (Figure 6a). As the whole process leading to lung collagen deposition takes time to build up, only the 28-day samples were analyzed. An open-access MATLAB-based image analysis software (see details in the experimental section) was used to determine the percentage of collagen-positive areas across the stained lung sections (Figure 6a, blue). A significant increase in collagen deposition in BNNT-exposed animals was found compared to that of the negative control or *h*-BN, suggesting ongoing lung fibrosis after exposure to BNNTs (Figure 6a).

Potential DNA damages (*i.e*., DNA double-strand breaks) in the lung parenchyma were also evaluated on day 28 using a monoclonal antibody targeting γ-H2AX. Lungs of mice exposed to methylmethanesulfonate (MMS) by gavage (3 times to 150 mg/kg; 48, 24, and 4 h before sacrifice) were used as a positive control for DNA damage. Exposure to high aspect ratio BNNTs caused a significant increase in the number of DNA double-strand breaks compared to the vehicle control, whereas low aspect ratio *h*-BN did not (Figure 6b). This was in line with previous studies in which MWCNTs with a high aspect ratio promoted a chronic inflammatory lung environment that could later induce persistent DNA damage.^26,27^

### Comparing the Pulmonary Impact of *h*-BN Nanosheets with Other 2D Materials

In the present study, it is assumed that a single dose of 30 μg per mouse was representative of a worst-case scenario such as accidental exposure at a production facility with no respiratory protection.^11,16^ Despite this high dose, low aspect ratio *h*-BN nanosheets did not cause any lung inflammation or damage and were eliminated efficiently from the lungs, with only a few materials remaining after 28 days. The absence of acute immune response was somewhat surprising because most of the 2D materials tested so far by us and others induced at least an acute lung inflammation after administration to high doses in rodents.^35,39,40^

In several studies, single pharyngeal aspiration of graphene nanoplatelets (GNPs) or few-layer graphene (FLGs) caused acute lung inflammation that resolved by day 7 or 28.^39,41−44^ Importantly, in most of these studies, the authors did not observe chronic inflammation or tissue damage, although some of these materials were shown to persist in the respiratory tract for a long period.^39,41−44^ In this respect, Schinwald et al., demonstrated that GNP biopersistence was primarily due to frustrated phagocytosis in lung macrophages.^41,44^ Compared with our findings with *h*-BN, it could be hypothesized that the acute inflammation reported in these studies may be linked to the biopersistence of these 2D materials in the lungs. Therefore, the absence of acute inflammation found here for *h*-BN could be due to its efficient and consistent elimination from the lung airways, as evidenced by Raman imaging. However, in other studies, after 5 or 28 days of continuous inhalation of GNPs, which represents a more physiological method of administration, no significant inflammation was reported after exposure, even at the highest dose tested and despite the materials being shown to persist in the lungs.^45,46^

Similarly to GNPs or FLGs, exposure to GO sheets was also found to induce acute lung inflammation in rodents.^47−49^ Bengston et al. further reported that reduced GO but not GO could cause chronic lung inflammation.^49^ Both materials were shown to be genotoxic in BAL immune cells but not in lung tissues.^49^ Importantly, the pulmonary toxicity of GBMs was shown to be size-dependent. In several studies, including ours, stronger inflammation profiles and slower recovery were reported for micrometric when compared to nanometric sheets (either GNPs or GO).^25−27,35,39,50,51^ In our previous work, the size-dependent differences were ascribed to impaired degradation of micrometric GO sheets in alveolar macrophages compared to nanometric GO sheets, which combined with a reduced clearance of the micrometric materials from the alveolar region led to their biopersistence in the lungs, and therefore a more adverse impact.^35^ Additionally, in a recent study specifically designed to evaluate the chronic immune response and impact after multiple challenges with GO sheets, we revealed that the absence of an adaptive immunity activation by GO with small lateral dimensions was a good prognosis of GBM lung biocompatibility and could explain the efficient recovery and absence of tissue damage.^26,27^ Herein, it could therefore be inferred that *h*-BN nanosheets did not cause inflammation not only because of their elimination from the airways but also because of their small lateral dimensions, which together with their relatively small thickness provide them with low aspect ratio physical characteristics.

While comparison with other 2D materials is somewhat facilitated by the abundance of studies, direct comparison with other *in vivo h*-BN findings is impossible due to the lack of such reports in the current literature. Nevertheless, Kodali et al., reported *in vitro* that *h*-BN could cause inflammation and toxicity in the macrophage-derived THP-1 cell line, albeit to a lower extent compared to BNNTs.^18^ In another study, Xu et al., evaluated the toxicity of *h*-BN and several 2D transition metal dichalcogenides on the broncho-epithelial BEAS-2B immortalized cells and macrophage-derived THP-1 cell line.^52^ The authors did not observe any toxicity for *h*-BN but reported toxicity for MoS_2_ and WS_2_. They further explored the impact of MoS_2_ and WS_2_ after pharyngeal aspiration in mice and reported acute lung inflammation that could be alleviated by the nanomaterials surface passivation, but unfortunately they did not evaluate the *in vivo* impact of their *h*-BN nanosheets.^52^

Bringing together the findings of the aforementioned research on 2D materials and the current results, it can be concluded that the low aspect ratio *h*-BN nanosheets tested herein appear safer for the lungs than most of the 2D materials tested so far.^35,39,41−44^ Nevertheless, we must highlight that we tested *h*-BN with defined morphological properties. Thus, testing *h*-BN of different thicknesses, lateral dimensions, shapes, or preparation using other exfoliation agents or methodologies appears important to assess. For instance, it has been recently demonstrated that *h*-BN prepared with either rhomboidal, cornered morphology, or a round morphology could achieve different toxicological outcomes in the lung epithelial H460 cell line.^17^ In this study, cornered *h*-BN induced dose-dependent cytotoxicity and apoptosis, while rounded *h*-BN nanosheets (which resemble the *h*-BN used here) did not cause any effect. Going further, it would therefore be interesting to assess the toxicity of all possible *h*-BN derivatives using alternative methods such as advanced human lung cell models to perform the toxicological assessment of these materials in a high throughput fashion without increasing the number of animals used in research.^53^ Before any definitive conclusion is drawn about the potential safety of *h*-BN materials after lung administration, it will also be necessary to assess the impact of chronic exposure to these materials in these advanced cell models or animals.

### BNNT Impact in Lungs in Comparison to Other High Aspect Ratio Materials

Herein, exposure of mice to high aspect ratio BNNTs caused significant lung adverse effects for up to 28 days. Both acute and chronic inflammation was induced, with markers and cells linked to the activation of both innate and adaptive immunity. Furthermore, the study revealed evidence of tissue remodeling (i.e., fibrosis) and lung damage. This included an increase in the bronchial thickness, collagen deposition, and DNA damage in the lung parenchyma. Such extensive adverse effects are likely to be ascribed to the poor elimination of the BNNTs from the lungs due to ongoing frustrated phagocytosis in lung macrophages, and contrast with the findings for low aspect ratio *h*-BN nanosheets that appeared to be removed efficiently from the airways. Overall, the striking contrast between high aspect ratio BNNTs and low aspect ratio *h*-BN effects highlights their main physical difference, *i.e.*, their dimensional aspect ratio, as the leading cause of their toxicity, in a similar fashion to what was reported before for carbon-based materials when comparing long and rigid carbon nanotubes with small dimension graphene nanoplatelets or GO.^26,27,35^

In a comparable study to ours, Xin et al. reported acute and chronic adverse effects after pharyngeal aspiration to 40 μg of BNNTs.^11^ In line with our findings, they found an influx in neutrophils and eosinophils as well as the presence of lymphocytes in BALF.^11^ They also described an increase in inflammatory markers of the innate immunity, up to 2 months after exposure, and of the adaptive immunity up to 7 days after exposure.^11^ Their results suggested that BNNTs, in a similar fashion to long and rigid MWCNTs,^26^ induced an immunogenic-like response. Indeed, BNNTs were inducing a significant variation in the lymphocyte populations in the lung-draining lymph nodes.^11^ Moreover, and in agreement with our results, they reported a poor clearance of the BNNTs from the lungs, with more than 50% of the materials remaining after 2 months.^11^ However, despite this persistence in the lungs, and in contrast to our results, they did not observe tissue damage or fibrosis.^11^ This is surprising because the authors highlighted the strong similarities between the lung response to the BNNTs they used and the response reported before for long and rigid MWCNTs, which were shown to cause chronic lung inflammation, fibrosis, and tissue damage.^10,11,16^ As part of this response to BNNTs, they reported the activation of similar adverse outcome pathways to those activated by MWCNTs, including the inflammasome NLRP3, and the chronic expression of several inflammatory markers,^11^ including osteopontin, a marker typically involved in granuloma formation and fibrosis. The authors hypothesized that this discrepancy (*i.e*., not inducing fibrosis despite inducing fibrosis markers) could be attributed to the low nanotube content of the BNNTs used because they were composed of only 50% of nanotubes by mass.^11^ In our work, the BNNTs had less than 1% elemental boron and between 80 and 90% of nanotubes based on our TEM images. In comparison to Xin et al.,^11^ this higher amount of nanotubes per administrated dose (for a similar dose: 40 μg in Xin et al.^11^ vs 30 μg here) may explain our findings and the 28-day lung remodeling and DNA damage, which are both in agreement with previous works testing long and rigid MWCNTs.^13,26,27^

Due to their similar physical characteristics (*i.e.*, high aspect ratio), it was indeed expected that the long BNNTs used here would show a biological response comparable to what was reported before for long and rigid MWCNTs, including hallmarks of the fiber pathogenicity paradigm. In contrast, *h-*BN nanosheets due to their low aspect ratio were not expected to present any similarities to MWCNTs in the response they induce in the lungs. According to the fiber pathogenicity paradigm, the toxicity of respirable fibers is dependent on their length and width, with nanofibers above 10 μm in length inducing frustrated phagocytosis in lung macrophages, granulomas, and fibrosis, as well as further damage in the pleura, and even mesothelioma if they translocate from the airways into the pleural cavity.^20,22^ This has been demonstrated in several studies using long MWCNTs^19,20,36,54^ or other nanofibers such as silver nanowires.^55^ In the case of MWCNTs, they even reported that the nanotubes were able to translocate into the pleural cavity and cause further tissue damage therein.^56,57^ In the present study, we revealed the occurrence of BNNT frustrated phagocytosis in alveolar macrophages, alveolar retention of BNNTs, and the induction of chronic inflammation and lung damage but found no clear evidence of BNNTs presence in the pleural cavity. However, we measured an increase in pleural thickness that may suggest a potential impact of the BNNTs on the pleural epithelium, warranting further work to evaluate the ability of BNNTs to translocate into the pleural cavity, as previously described for MWCNTs.^56,57^

According to the fiber pathogenicity paradigm, a length preventing a good clearance from the airways leading to material biopersistence in the lungs and formation of granulomatous structures is likely to cause fibrotic lesions and long-term damage.^58^ Moreover, the activation of the adaptive immunity (Th1/Th2), which was found here for BNNTs but not for *h*-BN nanosheets, is a well-known underpinning biological mechanism that plays a role in both fibrosis and DNA damage following exposure to high aspect ratio nanomaterials.^13,59−61^ Therefore, the main physical characteristic of the BNNTs, their long length in respect to their narrow diameter (*i.e.*, a length between 2 and >10 μm for a 2–4 nm diameter) is likely the main driver of their lung pathogenicity, as previously reported for long MWCNTs. Future studies should aim at testing whether shorter BNNTs may fail to activate the adaptive immunity and therefore lose their ability to damage lungs, in line with earlier reports for shortened MWCNTs when compared to long MWCNTs.^23,62^

Taking into consideration both the present results for BNNTs and *h*-BN nanosheets and the wider literature regarding carbon nanotubes and graphene platelets, it can be inferred that irrespective of their chemical nature, high aspect ratio nanomaterials that conform to the fiber pathogenicity paradigm are very likely to induce lung pathogenicity, whereas low aspect ratio nanomaterials will have a limited pulmonary impact. Going further, this suggests that 2D materials may by default present a lower risk of pulmonary toxicity than 1D nanomaterials, unless their lateral dimensions reach a point at which they might be considered high aspect ratio 2D materials or are made of atoms with known toxicity. On the other hand, the present findings further confirm that high aspect ratio, tubular-shaped, nanomaterials should always raise safety concerns for lung health, regardless of their chemical nature, especially when these materials do not biodegrade. This outcome is likely something to take into consideration for future advanced materials or materials of emerging concerns such as polymer-based nanofibers.

## Conclusion

In the present work, we evaluated and compared the pulmonary toxicity of two boron nitride nanomaterials, namely, 2D nanosheets (*h*-BN) and 1D nanotubes (BNNTs), in mice after oro-pharyngeal aspiration. Low aspect ratio *h*-BN nanosheets were found to be safe for the lungs under the tested conditions, while high aspect ratio BNNTs caused significant adverse effects. In detail, *h*-BN nanosheets did not induce inflammation at any of the time points tested and were eliminated from the lung airways in a time-dependent fashion. On the contrary, BNNTs induced both acute and chronic inflammation, activated both innate and adaptive immunity, and led to granulomatous structures. Moreover, BNNTs had poor lung clearance and formed persistent material agglomerates that grew in dimension with time and were surrounded by immune cells. Because of their biopersistence and chronic activation of immune responses, BNNTs further induced pulmonary fibrosis and DNA double-strand breaks at the latest time point tested. These latter worrying effects are evidence of the potential of BNNTs to cause long-term effects and eventually chronic pulmonary diseases, as previously reported for other high aspect ratio nanomaterials such as long multiwalled carbon nanotubes. In summary, this work on boron nitride nanomaterials, along with previous studies on carbon nanomaterials, suggests that low aspect ratio 2D nanomaterials are overall safer than their high aspect ratio 1D nanotube counterparts, irrespective of their chemical nature. It further confirms that the dimensional aspect ratio of nanomaterials is an important if not the main factor leading to their lung pathogenicity. Overall, this work contributes to a better understanding of the safety profile of 2D materials and emphasizes the need to apply the most stringent control measures when manipulating high aspect ratio nanomaterials that conform to the fiber pathogenicity paradigm, regardless of their chemical composition, to prevent human exposure and the associated pulmonary consequences.

## Experimental Section

### Materials Production

Two-dimensional hexagonal boron nitride nanosheets were provided by BeDimensional SpA. The liquid-phase exfoliation process used to generate the *h*-BN suspension has been previously reported.^63,64^ In brief, the bulk *h*-BN is dispersed in distilled water and sodium cholate (10 wt %, Merck-Sigma), and then the mixture is agitated by using a mechanical stirrer at 500 rpm until no lumps are observed. Finally, the mixture is processed in a high-pressure homogenizer at 200 MPa.^63,64^

BNNTs (refined puffball SP10RX) were synthesized by BNNT LLC (Newport News, VA), via the high-temperature–pressure method (HTP).^65^ BNNTs were purified (99 wt % with 80–90% of the materials in the form of nanotubes, the remaining being *h*-BN sheets) *via* a high-temperature steam purification process to remove non-nanotube BN species as described in U.S. Patent US11629054B2, titled *Boron Nitride Nanotube Purification*, published on 2023-08-18. BNNTs were provided as puff balls that were first dispersed in absolute ethanol (bath sonication) before being thoroughly washed with milli-Q water to prepare a starting suspension at 2 mg/mL.

### Materials Characterization

Transmission electron microscopy analyses of *h*-BN and BNNTs were performed on a Hitachi 7500 transmission electron microscope (Hitachi High Technologies Corporation, Japan) equipped with an AMT Hamamatsu digital camera (Hamamatsu Photonics, Japan) and on a MET JEOL JEM 1400 ORIUS, respectively. Highly diluted samples were deposited on Formvar grids and dried before observation. Both *h*-BN and BNNTs were drop cast on mica discs and then analyzed using atomic force microscopy (AFM) (Bruker MultiMode 8) equipped with NanoScope v1.9 Software for the analysis (Figure 1).

Thermogravimetric analysis was performed on a TGA1 (Mettler Toledo) apparatus from 30 to 900 °C with a ramp of 10 °C/min under N_2_ using a flow rate of 50 mL/min and platinum pans. Samples were lyophilized before analysis.

Raman spectra of *h*-BN and BNNTs drop cast on quartz slides (Electron Microscopy Sciences, Inc.) were acquired using a Raman confocal system (XploRA Plus, HORIBA), equipped with a 638 nm LASER (slit at 100 and hole at 100) (SI, Figure S1).

X-ray photoelectron spectroscopy analysis was performed on a Thermo Scientific K X-ray photoelectron spectrometer with a basic chamber pressure of 10^–8^–10^–9^ bar and an Al anode as the X-ray source (1486 eV). The samples were analyzed as powder pressed onto a Scotch brand tape (3MTM EMI Copper Foil Shielding Tape 118). A spot size of 400 μm was used for analysis. The survey spectra are an average of 10 scans with a pass energy of 200.00 eV and a step size of 1 eV. For each sample, the analysis was repeated three times. A flood gun was turned on during the analysis. For data analysis, casaXPS (2.3.18) software was used. A Shirley background subtraction was applied. A line shape of 70% Gaussian/30% Lorenzian [GL (30)] was selected for all peaks (SI, Figure S2).

### Animal Exposure

Six week old C57BL/6J female mice were purchased from Envigo, UK. The mice treatment was randomized, and animals were kept in groups of four in ventilated cages with *ad libitum* access to food and water in a controlled environment (humidity, temperature, and light). All procedures were conducted after ethical approval from the UK Home Office, under Project License no. P089E2E0A. Following a week of acclimatization, the animals were exposed to a solution of 1 μg/μL (30 μg of materials in 30 μL of 0.5 % bovine serum albumin (BSA; Gibco, ThermoFisherScientific) in water for injection (v/v)) or controls (negative: vehicle, *Pseudomonas aeruginosa*; Merck-Sigma) 0.5 mg/kg) by single oropharyngeal aspiration. For the procedure, the mice were anesthetized by inhalation of 3% isoflurane in 100% oxygen and then held on a slanted board to deliver the materials or controls. The animals (*n* = 5) were kept for 1, 7, or 28 days after exposure. LPS 0.5 mg/kg delivered via oropharyngeal aspiration was used as a positive control to induce acute inflammation. Methylmethanesulfonate (MMS; Merck-Sigma; 3 × 150 mg/kg; *n* = 3; provided by gavage, 48, 24, and 4 h before sacrifice) was used as a positive control for DNA damage.

### Collection of the Samples

After the exposure, at the due time (1, 7, or 28 days), the animals were euthanized by IP injection of pentobarbitone. The left lung was clamped, and the right lung was washed with ice-cold PBS (Merck-Sigma) to collect BAL cells (*n* = 5). The right lungs were then cut and frozen in liquid nitrogen and stored at −80 °C for ELISA or RT-qPCR analysis (*n* = 5). Nonwashed left lungs were inflated with formalin to avoid alveolar collapsing and then kept in 10% formalin (Merck-Sigma) for 24 h (*n* = 5).

### Evaluation of Lung Clearance Using Raman Spectroscopy

Lungs stored in 10% formalin (Merck-Sigma) for 24 h were transferred to vials containing 70% ethanol (ThermoFisherScientific). The lungs were embedded in paraffin, and sections of 5.0 μm thickness were obtained using a microtome (RM2255, Leica Biosystems).

Xylene deparaffinized lung sections were scanned by Raman microscopy (XploRA Plus, HORIBA) using a laser excitation wavelength of 638 nm and a grating of 600, with 3 μm of the distance between each point. *h*-BN and BNNTs were found using the typical Raman shift present at 1370 cm^–1^. The nanomaterials were localized in the lungs by overlapping the images of Raman positive pixels and bright-field images of the scanned areas. The clearance over time was evaluated by scanning lung sections on days 1, 7, and 28 after the exposure.

### BAL Fluid Analysis

The collected BAL fluids were stored on ice and then centrifuged at 1500 rpm for 5 min at 4 °C (Hettich GmbH). The supernatants were aliquoted and stored at −20 °C and the cell pellets were suspended in PBS (Merck-Sigma). After counting, the cells were cytospun at 600 rpm (Hettich GmbH) for 5 min on superfrost plus slides (Epedia) with ∼100 000 cells/slide. The slides were then fixed in 100% ice-cold methanol for 10 min and then stored at −20 °C. The slides were stained using the Kwik-Diff kit (Shandon, ThermoFisherScientific) following the provider’s instructions, and the numbers of neutrophils, eosinophils, macrophages, and lymphocytes were determined after acquiring bright-field images (Pannoramic 250 Flash, 3D Histech Ltd.).

### Evaluation of the Inflammation Using Multiplex ELISA

The collected right lungs were digested in 1 mL of RIPA buffer (Merck-Sigma) supplemented with EDTA-free protease inhibitor (complete Mini, Roche) and homogenized (10 min at 50 Hz) using 5 mm stainless steel beads in a Tissue Lyser (Qiagen). Lysates were centrifuged for 5 min at 2600*g* (Hettich GmbH), and supernatants were stored at −80 °C until analysis. The total protein contents were measured using a BCA assay (Pierce, Thermo Fisher Scientific) and IL-1α, IL-1b, IL-6, TNF-α, MCP-1, GM-CSF, IL-17A, IL-23, IL-12p70, IFN-γ, IFN-β, IL-27, and IL-10 concentrations were assessed using a multiplex Mouse Inflammation Panel (13-plex, v-plate, Biolegend) according to the manufacturer’s instructions and using a BD FACS Verse flow cytometer (BD Biosciences). The concentration of each sample was determined using standard curves. IL-4 concentration in lysed lungs was determined using a single ELISA kit (mouse IL-4, Biolegend). Cytokine concentrations were expressed in pg/mg of protein by normalizing the obtained concentration to the total protein measured using the BCA assay.

### Gene Expression by RT-qPCR

The lungs kept at −80 °C were thawed and lysed in 1 mL of lysis buffer containing 2-mercaptoethanol (Gibco, ThermoFisherScientific) using a tissue lyser homogenizer running at 50 Hz for 10 min (Qiagen) with 5 mm stainless steel beads. Lysed lungs were centrifuged at 2600*g* for 5 min (Hettich GmbH) to remove cell debris, and supernatants were kept at −80 °C before extraction. Then, RNA was extracted using spin cartridges (PureLink RNA Mini kit, Qiagen) according to the manufacturer’s instructions. Total RNA concentration and purity were measured using a NanoDrop (ThermoFisherScientitific). First-strand cDNA was produced from 1 μg of extracted RNA using a High-Capacity cDNA Reverse Transcription kit (Applied Biosystems). Then 2 μL of cDNA obtained from reverse transcription were mixed with primers (Merck-Sigma; SI, Table S2) at 500 nM, and 10 μL of PowerUp SYBR Green Master Mix (Applied Biosystems, ThermoFisherScientific) in a 20 μL reaction. The PCR reaction (Biorad) consisted of a first activation step at 50 °C for 2 min, followed by a denaturation step at 95 °C for 2 min, before 40 cycles of amplification (denaturation at 95 °C for 15 s, then annealing, and elongation at 60 °C for 1 min). The ΔΔ*Ct* values of each sample were calculated, normalized to housekeeping gene values, and then expressed in fold change compared to the negative control (i.e., vehicle-treated animals).

### Histopathology of the Lungs

For histopathological analysis, sections were stained with hematoxylin and eosin using an automatic stainer (XL autostainer, Leica Biosystems) after being deparaffinized using subsequent baths of xylene, ethanol, and water. Bright-field images were generated with a slide scanner (Pannoramic 250 Flash, 3DHistech Ltd.). For each lung section, pleural and bronchial thickness, granulomatous, and immune cell infiltration areas were measured and analyzed using CaseViewer (version 2.4.0.11902, 3D Histech Ltd.).

### Evaluation of Collagen Deposition in the Lung Parenchyma

After deparaffinization using subsequent baths of xylene, ethanol, and water, lung sections were stained with Masson’s Trichrome (28-day samples only) to evaluate the potential collagen deposition as a marker of fibrosis. Collagen deposition was determined using a custom-made MATLAB program previously developed by Gutruf et al.^66^ The code uses color deconvolution to quantify the percent volume of fibrosis in images of lung sections stained with Masson’s Trichrome (https://github.com/optocardiography/massonstrichromequantification).^66^

### Evaluation of Potential Genotoxicity in the Lung Parenchyma Using γ-H2AX

The level of DNA damage was evaluated on formalin-fixed, paraffin-embedded lung sections using immunofluorescence. A rabbit anti-mouse γ-H2A.X recombinant antibody (BLR053F, ab243906; Abcam) and a donkey anti-rabbit secondary antibody (Alexa fluor 647, A-31573, Thermo Fisher Scientific) were used for staining potential DNA strand-breaks in the cell nucleus.^27^

Briefly, lung sections were deparaffinized using subsequent baths with xylene, ethanol, and water. Lung sections were heated in the microwave in a citrate buffer (10 mM sodium citrate, 0.05% tween 20; pH = 6) for antigen retrieval. Then, the sections were permeated with 0.1% Triton X-100 and unspecific antigens were blocked for 1 h using 10% normal donkey serum and 1% BSA. Lungs were incubated overnight at 4 °C with the primary antibody (γ-H2AX, 1/250 dilution), followed by 1 h incubation with the secondary antibody (1/400 dilution), both suspended in 1% BSA in PBS. The sections were finally mounted using mounting medium (ProLong Gold Antifade Mountant with DAPI, ThermoFisherScientific) and glass coverslips.

Nonstained sections were prepared simultaneously and used for background noise removal. Fourteen images per animal, from different parts of the lungs, were acquired with DAPI and Cy5 filters using a Zeiss AxioImager.D2 upright fluorescence microscope (Zeiss) and a 20×/0.5 EC Plan-neofluoar objective. For analysis, we recorded the number of γ-H2AX positive nuclei for every animal. Results were compared to the negative control (vehicle-treated animals) and positive control (mice exposed three times by gavage to methylmethanesulfonate (MMS): 3 × 150 mg/kg, 48, 24, and 4 h before sacrifice, *n* = 3). We focused solely on the 28-day samples to examine if there were any long-term DNA damage in the lung tissue due to exposure to boron nanomaterials.

### Statistical Analysis

Data were expressed as mean ± standard deviation. GraphPad Prism 9.0 (GraphPad Software Inc., San Diego, CA) was used to evaluate potential statistical differences. Two-way ANOVA followed by Dunnett’s posthoc test was used for BAL analysis (*n* = 5; *p* < 0.05 (*), *p* < 0.01 (**), *p* < 0.001 (***)). For ELISA and PCR analysis, one-way ANOVA followed by Dunnett posthoc test was used to evaluate differences in comparison to the negative control (*n* = 5; *p* < 0.05 (*), *p* < 0.01 (**), *p* < 0.001 (***)). Two-way ANOVA followed by Dunnett’s posthoc test was used for the histopathological analysis of the lung structures (*n* = 5; *p* < 0.05 (*), *p* < 0.01 (**), *p* < 0.001 (***)). Differences in collagen deposition were evaluated on Masson Trichrome stained slides using one-way ANOVA followed by Kruskal–Wallis posthoc test to evaluate significant differences *n* = 5, a minimum of 5 images/animal; *p* < 0.05 (*), *p* < 0.01 (**), *p* < 0.001 (***)). For DNA damage evaluation, one-way ANOVA followed by the Kruskal–Wallis posthoc test was used to evaluate statistical differences between *h*-BN, BNNTs, MMS (positive control), and the negative control (*n* = 3–5, minimum of 7 images/animal; *p* < 0.05 (*), *p* < 0.01 (**), *p* < 0.001 (***)).

## Supporting Information Available

Numerical results of XPS survey; RT-qPCR primers; physicochemical characteristics of BN materials; Raman, TGA, XPS analysis of the materials; representative pictures of BAL cells cytospun and differentially stained; AA protein measurement in blood samples (PDF)
