# Supplementary material for: Pulmonary Toxicity of Boron Nitride Nanomaterials Is Aspect Ratio Dependent
Source: ACS Nano. 2023 Dec 5;17(24):24919–35. doi: 10.1021/acsnano.3c06599 (PMC10753895; doi:10.1021/acsnano.3c06599)
Supplement: Supplementary file 1 — nn3c06599_si_001.pdf [file nn3c06599_si_001.pdf]

SUPPORTING INFORMATION for PUBLICATION

Pulmonary toxicity of boron nitride nanomaterials is aspect ratio dependent

Luis Augusto Visani de Luna[1,2,3^], Thomas Loret[1,2,3^], Yilin He[4], Morgan Legnani[5], Hazel Lin[4], Anne Marie Galibert[5], Alexander Fordham[1,2,3], Sonja Holme[1,2,3], Antonio Esau Del Rio Castillo[6], Francesco Bonaccorso[6,7], Alberto Bianco[4*], Emmanuel Flahaut[5*], Kostas Kostarelos[1,2,8*] and Cyrill Bussy[1,2,3,*]

[1] Nanomedicine Lab, Faculty of Biology, Medicine and Health, The University of Manchester, Manchester Academic Health Science Centre, Manchester, M13 9PT, UK
[2] National Graphene Institute, The University of Manchester, Manchester, M13 9PL, UK
[3] Lydia Becker Institute of Immunology and Inflammation, Faculty of Biology, Medicine and Health, The University of Manchester, Manchester Academic Health Science Centre, Manchester, M13 9PT, UK
[4] CNRS, Immunology, Immunopathology and Therapeutic Chemistry, UPR 3572, University of Strasbourg, ISIS, 67000 Strasbourg, France
[5] CIRIMAT, Université Toulouse 3 Paul Sabatier, Toulouse INP, CNRS, Université de Toulouse, 118 Route de Narbonne, 31062 Toulouse cedex 9, France
[6] BeDimensional S.p.A., Lungo Torrente Secca 30r, 16163 Genoa, Italy
[7] Istituto Italiano di Tecnologia, Graphene Labs, Via Morego 30, 16163 Genoa, Italy
[8] Catalan Institute of Nanoscience and Nanotechnology (ICN2), CSIC and BIST, Campus UAB, Bellaterra, 08193 Barcelona, Spain

---

^These authors contributed equally to this work

*Correspondence to:

a.bianco@ibmc-cnrs.unistra.fr;

emmanuel.flahaut@univ-tlse3.fr;

kostas.kostarelos@manchester.ac.uk;

cyrill.bussy@manchester.ac.uk

SUPPLEMENTARY TABLES

Table S1. Atomic ratio of *h*-BN and BNNTs calculated by XPS survey spectra.

	Atomic ratio (%)				
	B	N	C	O	Na
***h*-BN**	43.1	34.1	18.3	4.0	0.5
**BNNTs**	47.0	28.6	12.0	2.3	0.1

Table S2. Primers used for RT-qPCR.

	Forward	Reverse
**SAA-3**	AACTATGATGCTGCCCGGAG	GCTCCATGTCCCGTGAACTT
**Arginase-1**	CTTGCGAGACGTAGACCCTG	TGAGTTCCGAAGCAAGCCAA
**Osteopontin**	CTGGCAGCTCAGAGGAGAAG	ACAGGGATGACATCGAGGGA

Table S3. Summary of tested BN nanomaterials including method of synthesis, physico-chemical characterization, dose, exposure model and lung response.

	Method of synthesis	Summary of characterization	Tested dose	Exposure model	Lung response
***h*-BN**	liquid-phase exfoliation in sodium cholate	Size: ~290 nm (TEM)<br>Thickness: ~3.7 nm (AFM)<br>Composition: ~8% sodium cholate, 92% B, N, C, O	30 µg<br>In 0.5% BSA in water	Oro-pharyngeal aspiration in C57BL/6J female mice (n=5)	• Rapid clearance up 7d<br>• No inflammation (BALF cells)<br>• No inflammatory cytokine<br>• No tissue morphological changes<br>• No tissue fibrosis<br>• No DNA damage (γH2AX$^+$ cells)
**BNNT**	high-temperature-pressure method (HTP)	Diameter: 3 – 15 nm (TEM/AFM)<br>Length: > 5 µm<br>Composition: B, N, C, O			• Poor clearance at 28d<br>• Strong inflammation at 7d (BALF total cells)<br>• IL-1α, IL-1β, IL-6, TNF-α, MCP-1, GM-CSF, IL-17a, IL-27, SAA3 at 1d<br>• TNF-α, MCP-1, IL-17a, IFN-γ, IL-4, SAA3 and OPN at 7d<br>• IL-1α and IFN- γ, SAA3, Arg-1, at 28d<br>• Tissue morphological changes from 7d<br>• Tissue fibrosis at 28d<br>• DNA damage at 28d (γH2Ax positive cells)

BN: boron nitride nanomaterials; *h*-BN: hexagonal boron nitride; BNNT: boron nitride nanotubes; HTTP: high pressure temperature; TEM: transmission electron microscopy; AFM: atomic force microscopy; B: boron element; N: nitrogen; C: carbon; O: oxygen; BSA: bovine serum albumin; d: days; BALF: broncho-alveolar lavage fluid; IL: interleukin; IFN: interferon; SAA: serum amyloid A; OPN: osteopontin; Arg: arginase

SUPPLEMENTARY FIGURES

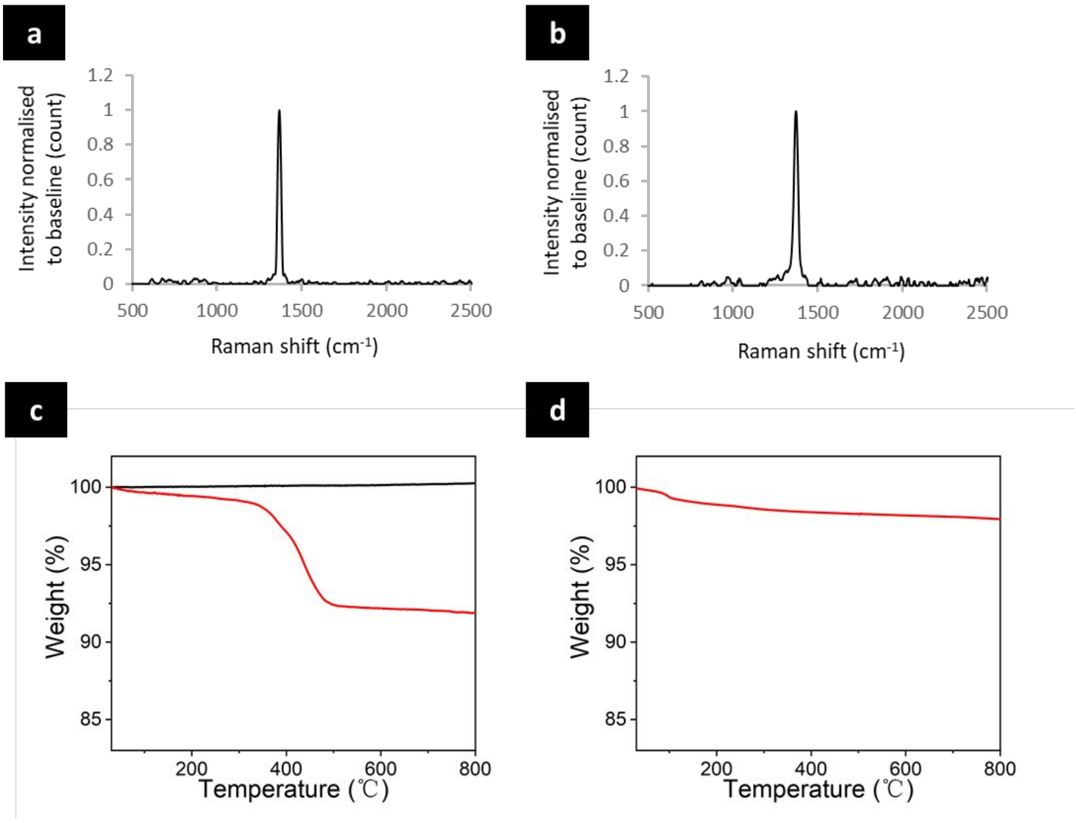

Figure S1. Structural characterization of *h*-BN and BNNTs. (a) and (b) Raman spectra of *h*-BN and BNNTs. (c) TGA curves of *h*-BN (black line: bulk *h*-BN, red line: exfoliated *h*-BN). (d) TGA curve of BNNTs.

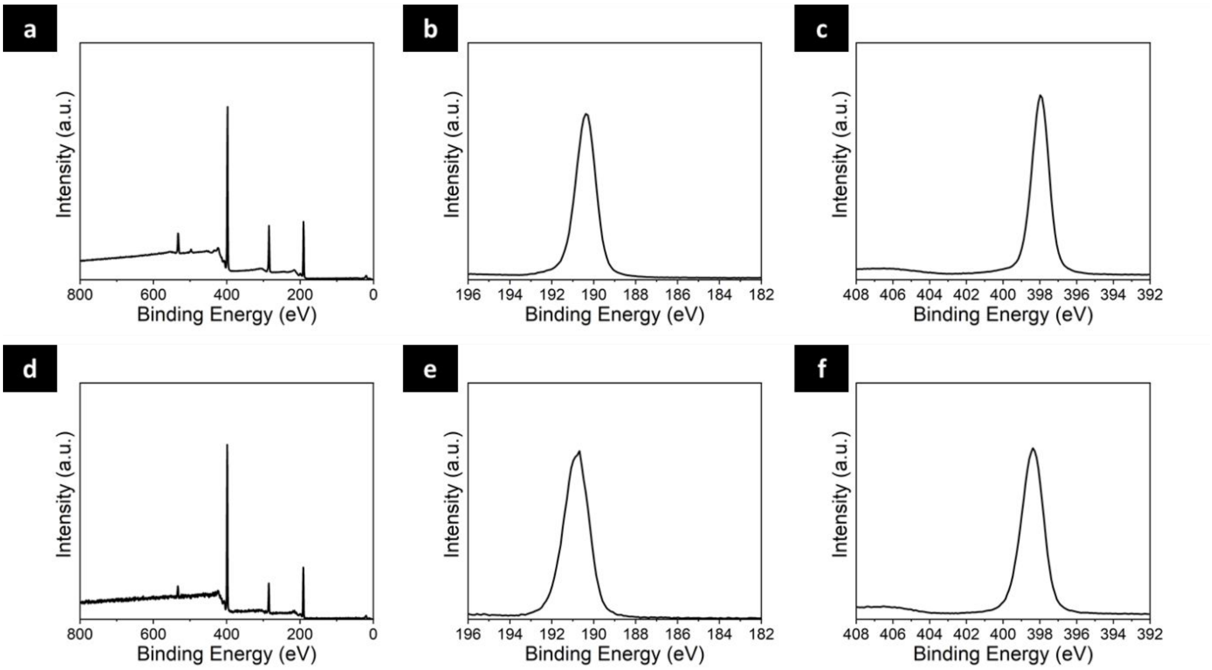

Figure S2. Structural characterization of *h*-BN and BNNTs. (a), (b) and (c) XPS survey, high-resolution B1s and N1s spectra of *h*-BN. (d), (e) and (f) XPS survey, high-resolution B1s and N1s spectra of BNNTs.

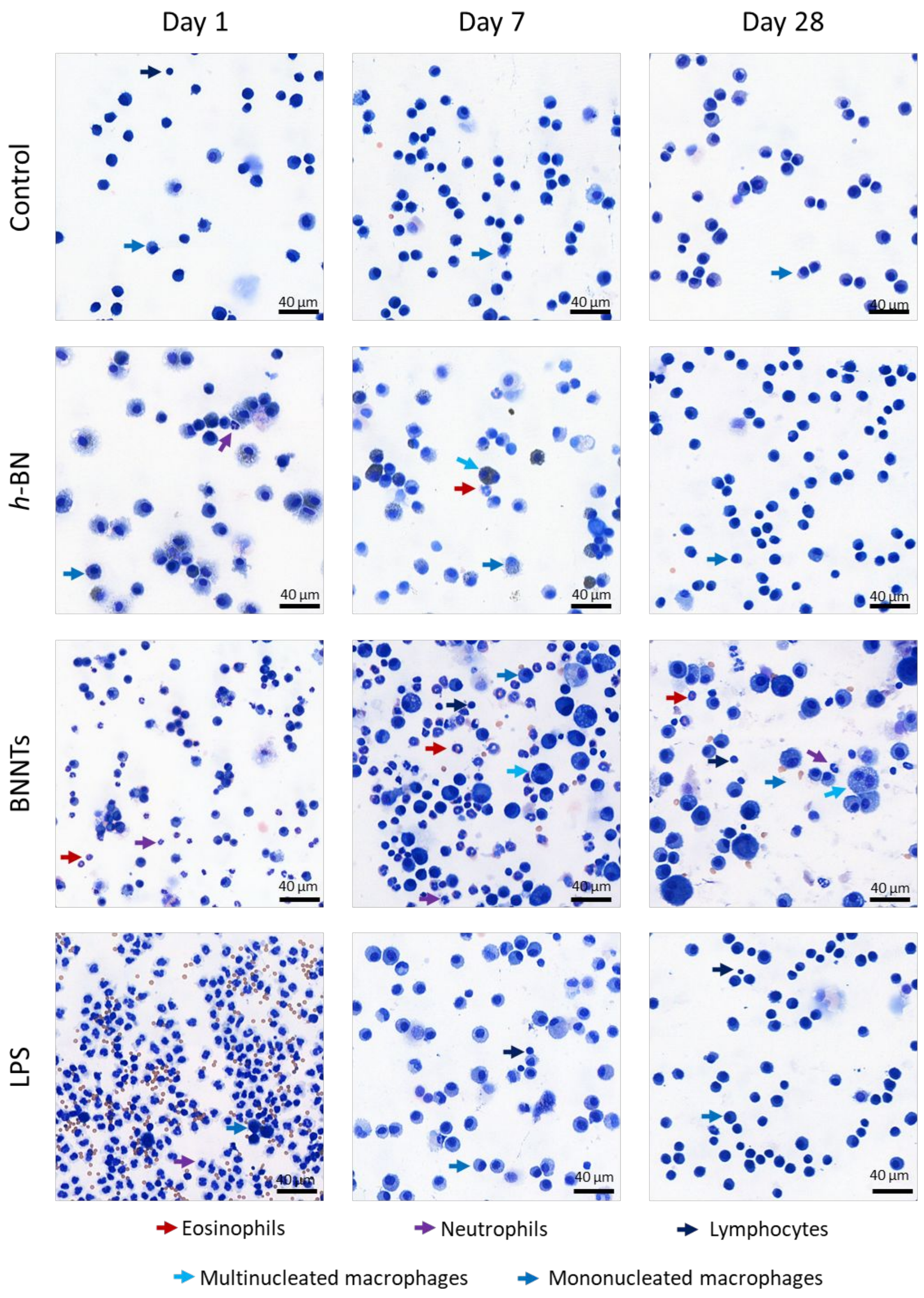

Figure S3. Influx of inflammatory cells in alveolar space. Mice were exposed by oropharyngeal aspiration to 30 µg of *h*-BN, BNNTs, or controls (vehicle control and LPS). BALFs were collected on days 1, 7, and 28, cyto-spun on slides and then stained for cell phenotyping using Diff-Quick staining.

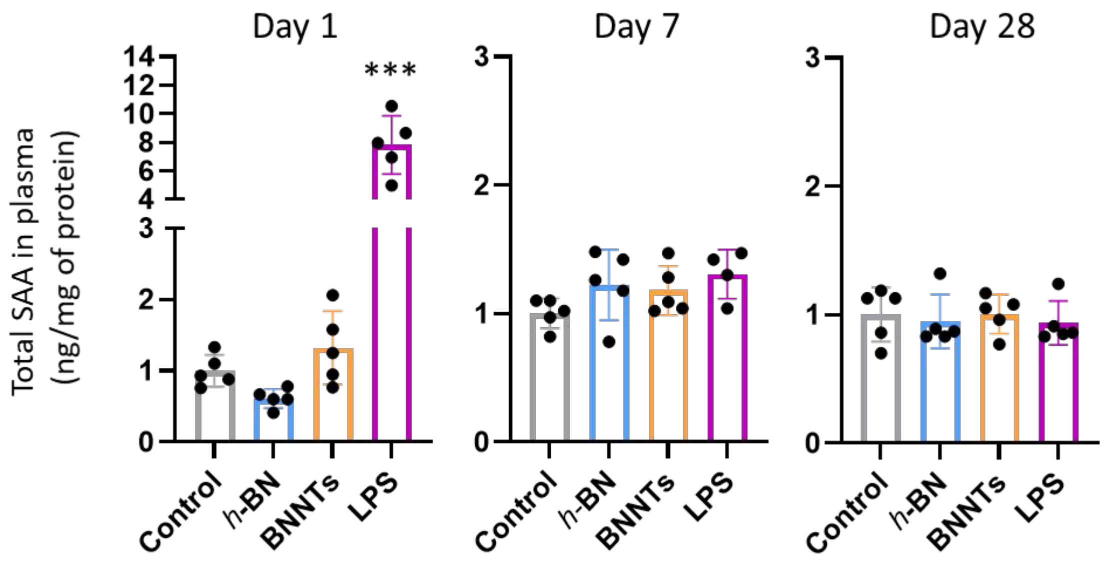

Figure S4. Acute response in blood samples. One-way ANOVA statistical analysis on the normalized cytokine concentrations compared to the negative control; pg/mg of total protein measured with BCA Pierce Assay. (*) $p<0.05$; (**) $p<0.01$; (***) $p<0.001$.
